# Supplementary figures and images for: Using drones and sirens to elicit avoidance behaviour in white rhinoceros as an anti-poaching tactic
Source: Proc Biol Sci. 2019 Jul 17;286(1907):20191135. doi: 10.1098/rspb.2019.1135 (PMC6661359; doi:10.1098/rspb.2019.1135)

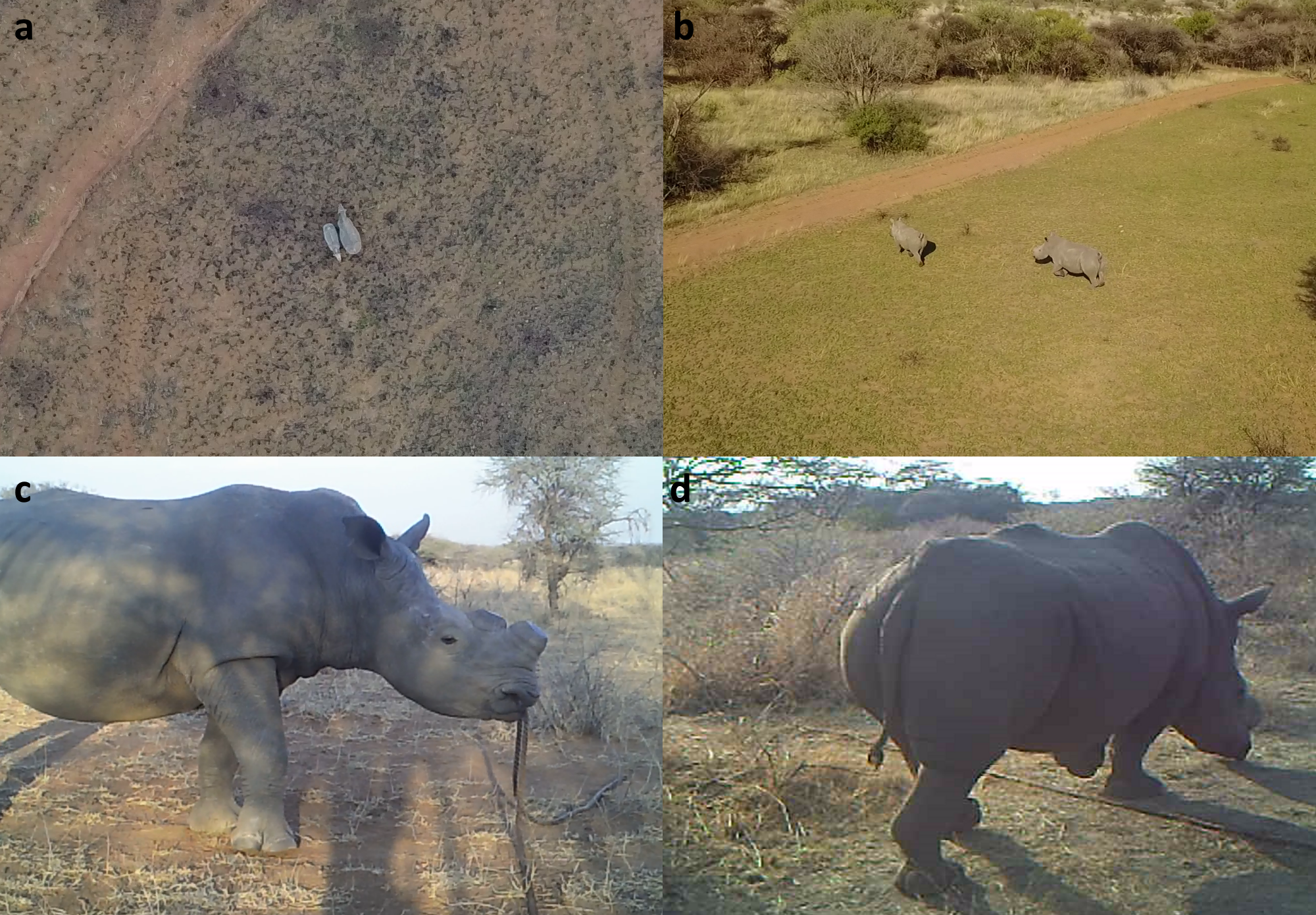

Supplement: Image panel showing white rhino behavioural responses to the drone and scent deterrent experiments. [file rspb20191135supp1.tif]
